# Effort-reward imbalance and quality of life of healthcare workers in military hospitals: a cross-sectional study

**DOI:** 10.1186/1472-6963-12-309

**Published:** 2012-09-08

**Authors:** Dong-Sheng Tzeng, Wei-Ching Chung, Chi-Hung Lin, Chun-Yuh Yang

**Affiliations:** 1 Department of Psychiatry, Kaohsiung Armed Forces Hospital, Kaohsiung, Taiwan; 2 Institute of Undersea and Hyperbaric Medicine, National Defense Medical Center, Taipei, Taiwan; 3 Department of Nursing, Fooyn General Hospital, Pingtung, Taiwan; 4 Faculty of Public Health, College of Health Sciences, Kaohsiung Medical University, Kaohsiung, Taiwan

**Keywords:** Effort-reward imbalance, GHQ, Overcommitment, WHOQOL-BREF

## Abstract

**Background:**

Taiwan’s National Defense Bureau has been merging its hospitals and adjusting hospital accreditation levels since the beginning of 2006. These changes have introduced many stressors to the healthcare workers in these hospitals. This study investigates the association between job stress, psychological morbidity and quality of life in healthcare workers in three military hospitals.

**Methods:**

We posted surveys to 1269 healthcare workers in three military hospitals located in southern Taiwan. The surveys included the General Health Questionnaire (GHQ), the World Health Organization Quality of Life Questionnaire (WHOQOL-BREF), and the Effort-Reward Imbalance (ERI) Questionnaire. High effort-reward (ER) ratio and overcommitment were defined when scores fell into the upper tertile of the total distribution.

**Results:**

The survey was completed by 791 healthcare workers. On average, women reported a higher ERI than men. High ERI was associated with younger age, higher psychological morbidity, and poor physical and psychological QOL domains in this population. High ER ratio and high overcommitment were associated with psychological morbidity and poor QOL in both sexes. However, high ER ratio was not significantly associated with the social QOL domain in either sexes or the physical QOL domain in males.

**Conclusions:**

There was a clear association between ERI and QOL in the healthcare workers in the military hospitals under reorganization and accreditation in this study. We found ER ratio and overcommitment to be suitable indicators of job stress.

## Background

Taiwan’s National Defense Medical Affairs Bureau has merged several of its hospitals and restructured many workplaces by ranking the hospitals according to their accreditation level. Resultantly, job insecurity and emotional distress may be serious problems in these workplaces [1]. The prevalence of psychological morbidity in the general and working population, as assessed by the General Health Questionnaire (GHQ), a generally accepted measure of emotional distress and mental health in occupational studies, ranges between 9% and 28% in men and between 21% and 33% in women [1-3].

Quality of life (QOL) is a broad-ranging concept, incorporating the person’s physical health, psychological state, level of independence, social relationships, personal beliefs and their relationship with salient features of the environment [4,5]. There are epidemiological studies on the QOL of healthcare workers in hospitals, where certain stressors influencing QOL can be found [1,6,7]. Social support has been studied as a possible predictor of health-related QOL [7]. Others have studied job stress in relation to general health [8], but do not cover all of the biological, psychological, social and environmental aspects of the World Health Organization (WHO) QOL definition.

Effort-reward imbalance (ERI) is a model developed to analyze job stress. It focuses on reciprocity of exchange in occupational life, in which high-cost/low-gain conditions are considered particularly stressful. In this model, there is explicit emphasis on individual attributes, particularly coping characteristics of high intrinsic effort, which are defined by the concept of “need for control” [9]. Extrinsic efforts, defined by a high workload, are also specified in the model. Three different sources of reward are taken into account: financial, esteem, and occupational social status control (i.e., promotion prospects and job security). Low occupational social status control, in relation to high extrinsic or intrinsic effort, has been shown to independently predict new cardiovascular events in a prospective study of blue-collar men [10]. ERI has been associated with a higher risk of coronary heart disease [11,12], while both ERI and overcommitment have been identified as correlates of salivary cortisol and higher blood pressure [13]. ERI has also been linked to other outcomes, including sick leave [14], alcohol dependence [15], psychological well-being [16], burnout and job satisfaction [17,18]. Moreover, ERI has been found to be predictive of General Health Questionnaire (GHQ) scores in men but not in women [3], depressive symptoms by clinical evaluation scale in men [2] and deleterious health by self-administered questionnaire in employed women [19].

This study examines the impact of different levels of ERI on biological, psychological, social and environmental QOL and on psychological morbidity among healthcare workers in military hospitals undergoing reorganization. This reorganization is undoubtedly a source of job stress for the healthcare workers serving in this healthcare system. We hypothesized that the healthcare workers with higher ERI would have higher psychological morbidity and lower QOL. We administered the Chinese versions of the GHQ, World Health Organization Quality of Life (WHOQOL) and ERI questionnaires and analyzed the association between poor QOL in the four domains, overcommitment and effort-reward ratio (ER ratio).

## Methods

This cross-sectional study was initiated in November 2005. We used purposive sampling to select personnel from three southern Taiwan military hospitals as study respondents. The Institutional Review Board in Kaohsiung general hospital approved this investigation and the respondents filled out a structured questionnaire with an informed consent form (IRB096IV004). After the researchers evaluated whether or not the respondents had any major diseases, healthcare workers willing to undergo the study filled out a self-report questionnaire. We excluded respondents with major diseases because the presence of disease would likely influence the outcome measurements.

### Respondents

The respondents were divided into three groups according to their occupation: physicians, nurses and other personnel (e.g., social workers, psychologists, pharmacologists, laboratory workers and executive officers). There were originally nineteen military hospitals in Taiwan. The Medical Affairs Bureau decided to merge them into nine hospitals, beginning in July 2006. We selected three hospitals to represent the Air Force, Navy and Armed Force in three different types of organizations. We used ‘Sample size determination in health studies’, version 2.0 (World Health Organization), selected the 95% confidence interval, anticipated population prevalence of 0.025, population size 50 000, and predicted the parameter of sample size to be 364. We enrolled 133 of 140 healthcare workers at the Air Force Pingtung Hospital; a response rate of 95%. Pingtung Hospital is a regional hospital with 3 acute wards and 100 beds, an emergency volume of 300 cases per month and a resource utilization of about 10 million Taiwan dollars per month. We enrolled 157 of 192 healthcare workers from the Navy Penghu Hospital; a response rate of 81.8%. Penghu Hospital is a regional hospital with 5 acute wards and 150 beds, an emergency volume of 600 cases per month and a resource utilization of about 15 million Taiwan dollars per month. The average work hours per week for physicians, nurses, specialized and administrative personnel for these two merged hospitals are 60, 36, 40 and 34, respectively We enrolled 501 of 937 healthcare workers from the Armed Force Kaohsiung General Hospital; a response rate of 53.5%. Kaohsiung General Hospital is a teaching hospital with 20 acute wards and 650 beds, 50 ICU beds, an emergency volume of 5500 cases per month and a resource utilization of about 150 million Taiwan dollars per month. In this hospital, the average work hours per week for physicians, nurses, specialized and administrative personnel are 78, 44, 42 and 34, respectively.

In total, we enrolled 791 of a potential 1269 healthcare workers; an overall response rate of 62.3%.

### Instruments

We anonymously collected each respondent’s basic information, including sex, age, job category, educational level, marital status, smoking history, history of alcohol consumption and hypnotic drug use, and life events in the past 6 months. Life events were defined as death of a relative, economic stress, marital or divorce event, sentinel events and malpractice problems with legal implications. These data are likely associated with the outcome measures. Questionnaires also included the GHQ, the Taiwan brief version of the WHO Quality of Life Questionnaire (WHOQOL-BREF), and the ERI Questionnaire.

The GHQ-12 is a self-administered screening instrument used to assess psychological morbidity in a Chinese community. It is the GHQ modified to include cultural items relevant to Chinese people in a primary item pool. Discriminant function analysis was used to select a subset of 12 items from the item pool. A simple scoring method was applied to the GHQ, with 0-0-1-1 representing the ratings "not at all" and "about as usual" as 0, "more than usual" and "strong feeling" as 1. Minimum to maximum score is 0 to 12. Psychological morbidity was first calculated using a global score, and then respondents were divided into potential cases and non-cases using an optimum cutoff point (the best compromise between high sensitivity and a low false-positive rate) of 2–3, based on Receiver Operating Characteristic curves. Cronbach’s alpha was 0.79 [20].

The WHOQOL-BREF is a revised version of the 100-item WHOQOL Questionnaire. The WHO developed this measure to evaluate QOL across countries and cultures. It has been used in many studies, including those of medical outcomes and health policies. The Taiwanese brief version of this measure, which has a total of 28 questions, has been modified to fit the local culture, and simplified and translated into Chinese. It is used to evaluate the respondent’s perceptions of his or her physical, psychological, social and environmental well-being during the four weeks prior to its administration. The higher the score, the higher the QOL was. The reliability test statistic between the WHOQOL-BREF and the original measure ranges from 0.70 to 0.80 and Cronbach’s alpha ranges from 0.70 to 0.77. For content validity, the Pearson’s correlation coefficient for each question and its measurement was between 0.53 and 0.78. Criteria-related validity can explain 60% of global QOL and construct validity can explain 73% of the variance by principal factor analysis [20]. QOL in any of the four domains was defined as poor if a respondent’s score fell within the range of the lowest tertile of the sample. The WHOQOL-BREF has good-to-excellent psychometric reliability, and has performed well in preliminary tests of validity [5]. These results indicate that overall, the WHOQOL-BREF is a sound, cross-culturally valid assessment of QOL [4].

The 23-item ERI questionnaire consists of three categories termed "effort" (6 items), "reward" (11 items, including esteem, job promotion and job security), and "overcommitment" (6 items). Responses to the "effort" and "reward" items are scored on a five-point scale, with 1 indicating no particularly stressful experience and 5 indicating a very highly stressful experience. The Cronbach’s alpha coefficients for "effort," "reward," and "overcommitment" for men are 0.80, 0.82, and 0.66, respectively; and for women are 0.83, 0.80 and 0.66 [21]. According to a predefined algorithm, a ratio between the two categories of "effort" and "reward" (weighted by item numbers) is calculated to quantify the degree of mismatch between high cost and low gain [22]. "Effort" and "reward" are coded as dichotomous variables using the median of the total sample as the cut point [22,23]. Positive effort-reward imbalance is defined by both high effort and low reward; negative effort-reward imbalance is defined by only high effort or low reward as well as by neither high effort nor low reward. High ER ratio and overcommitment are defined as falling within the upper tertile of the total distribution [23].

### Data analysis

Data were analyzed descriptively and then with chi-squared tests, independent sample *t*-tests and one-way ANOVAs. The chi-squared tests and independent sample *t*-tests were used to examine the differences in demographic variables and scales among male and female groups. The three levels of overcommitment and ER ratio were analyzed using one-way ANOVAs and logistic regression. Multiple regression analysis was used to test the association between ERI, psychological morbidity and QOL. A p-value < 0.05 was considered statistically significant. All statistical operations were performed using SPSS 10.0 for Windows (SPSS, Chicago, IL).

## Results

In total, 791 healthcare workers (66 physicians, 420 nurses, and 305 administrative and specialized personnel) from the three medical hospitals completed the survey. The lowest response rate was among physicians (37.5%), followed by administrative and specialized personnel (62.9%) and nurses (67.5%). The two smaller hospitals had high response rates (95% and 82%), while the larger hospital had a comparatively lower response rate (53%). The overall response rate was 62.3 %. The physicians were predominantly male (98.5%), and the nurses and others were predominantly female (98.8% and 65.8%, respectively). In women, we found a lower education level, less alcohol consumption, a lower prevalence of smoking history and fewer leadership positions compared with men (all p < 0.001). We also compared ERI, GHQ and WHOQOL scores between sexes. While women were found to have higher extrinsic effort scores than men (p = 0.003), there were no differences in intrinsic effort scores between the sexes. Men reported lower financial reward than women, while there was no significant sex difference in reported self-esteem reward. In women, we found a significantly lower social status control reward and lower total reward than in men, and their ER ratio was higher. There were no significant sex differences in scores on the four domains of the WHOQOL or in the total GHQ score (Table 1).

**Table 1:** Characteristics of study respondents, by sex (N = 791)

**Variables**	**Men (N = 173)**		**Women (N = 618)**			
	**Mean (N)**	**SD (%)**	**Mean (N)**	**SD (%)**	**t/X**^**2**^	**p**
Age (years)	35.8	7.5	35.1	9.0	0.83	0.408
Educational level (years)	16.5	2.3	14.1	1.7	15.27	<0.001***
Marital status					−1.83	0.068
Married	79	(46)	273	(44)		
Unmarried	61	(35)	193	(31)		
Other	1	(1)	26	(4)		
Life event					0.73	0.466
No	111	(64)	442	(72)		
Yes	14	(8)	44	(7)		
Alcohol drinking					7.70	<0.001***
No	103	(60)	478	(77)		
Yes	22	(13)	8	(2)		
Smoking					11.26	<0.001***
No	95	(55)	484	(78)		
Yes	31	(18)	4	(1)		
Hypnotics use					−0.17	0.869
No	123	(71)	479	(76)		
Yes	3	(2)	13	(2)		
Leader					5.36	<0.001***
No	144	(83)	588	(95)		
Yes	29	(17)	30	(5)		
ERI						
Effort	14.55	0.47	16.08	0.23	−2.66	0.003^**^
Reward	27.09	0.29	26.03	0.15	3.07	0.002^**^
Salary	1.65	0.09	1.94	0.06	−2.42	0.016^*^
Esteem	10.18	0.18	10.29	0.10	−0.48	0.634
Social Status Control	15.25	0.21	13.81	0.11	5.66	<0.001^***^
Overcommitment	16.04	0.22	16.22	0.11	−0.73	0.46
ER ratio	0.99	0.03	1.15	0.02	−3.92	<0.001^***^
GHQ	1.58	0.20	1.87	0.10	−1.36	0.19
WHOQOL-BREF						
Physical	25.4	0.43	25.5	0.16	−0.34	0.74
Psychological	19.7	0.34	19.5	0.15	0.76	0.45
Social	13.6	0.20	13.8	0.09	−1.21	0.23
Environmental	29.9	0.41	30.0	0.21	−0.18	0.85

*p < 0.05, **p < 0.01, ***p < 0.001.

We compared ERI scores between sexes (Table 2). Fourteen men (8.1%) had high ERI scores. While they had significantly more frequent life events than the low ERI group, and higher overcommitment scores (p < 0.001 and 0.009, respectively), they reported no greater psychological morbidity (p = 0.21), and their QOL scores in the physical, psychological, social and environmental domains of their QOL were similar to those of men with low ERI scores (p = 0.1 to 0.48). Forty-two women (6.8%) had high ERI scores. These women were significantly younger, had higher educational levels, a higher prevalence of hypnotic drug history, higher overcommitment scores and more psychological morbidity than women in the low ERI group (p < 0.001 to 0.037). While the physical and psychological QOL domain scores were worse in women with high ERI (p = 0.006 and 0.035, respectively), there were no differences in social or environmental domain scores between female ERI groups (p = 0.47 and 0.22, respectively).

**Table 2:** WHOQOL-BREF and GHQ scores by effort-reward imbalance group, by sex

**ERI**	**Male (N = 173)**			**Female (N = 618)**		
	**No (N = 159)**	**Yes (N = 14)**		** No (N = 576)**	**Yes (N = 42)**	
	**Mean**	**Mean**	**p**	**Mean**	**Mean**	**p**
Age	36.1	32.7	0.23	35.6	31.3	0.008**
Educational level	16.1	17.3	0.09	14.1	14.7	0.037*
overcommitment	15.9	17.9	0.009***	15.6	17.4	<0.001***
Total Score of GHQ	1.3	2.1	0.21	1.82	2.91	0.01**
WHOQOL-BREF						
Physical	25.6	23.3	0.21	25.7	23.9	0.006**
Psychological	19.9	18.9	0.48	19.5	18.2	0.035*
Social	13.6	13.7	0.10	13.8	13.6	0.47
Environmental	30.2	27.4	0.23	30.1	29.0	0.22

*p < 0.05, **p < 0.01, ***p < 0.001; ERI “yes” defined as both high effort and low reward, “no” defined as either high effort or low reward and neither high effort nor low reward.

As can be seen in Table 3, men who reported the highest ER ratios were more likely than men who reported low ER ratios to report psychological morbidity, and poor physical, psychological and environmental QOL. Men who reported the highest level of overcommitment were also more likely to report psychological morbidity, and poor psychological and environmental QOL than those who reported low levels of overcommitment. Women who reported intermediate or high ER ratios were more likely to report psychological morbidity, and poor physical, psychological and environmental QOL than women reporting the lowest ER ratio. No significant difference was found in social QOL between ER ratio groups. Women who reported intermediate or high overcommitment were more likely to report psychological morbidity, and poor physical, psychological, and environmental QOL than women who reported low overcommitment.

**Table 3:** Three regression analysis of relative factors associated with dependent variable as ER ratio, GHQ and total scale of QOL

	**R**	**R**^**2**^	**B**	**SE**	**β**	**95 % CI**		**p**
						**Lower bound**	**Upper bound**	
ER ratio	0.56	0.31						
Constant			0.11	0.13		−0.14	0.36	0.38
Sex			0.08	0.04	0.08	0.03	0.17	0.042
Age			−0.01	0.01	−0.15	−0.01	-0.04	<0.001
Reorganization or not			−0.06	0.03	−0.07	−0.12	-0.01	0.033
Nurse or not			0.09	0.04	0.12	0.03	0.17	0.006
GHQ	0.41	0.18						
Constant			−6.23	0.94		−8.07	-4.38	<0.001
Sex			0.39	0.23	0.07	−0.06	0.84	0.087
Life event			0.84	0.31	0.11	0.23	1.45	0.007
Hypnotic drug			1.09	0.61	0.07	−0.11	2.29	0.07
Overcommitment			0.28	0.04	0.30	0.20	0.36	<0.001
ER ratio			0.72	0.26	0.13	0.21	1.23	0.005
Total scale of QOL	0.48	0.23						
Constant			58.93	2.24		54.54	63.32	<0.001
GHQ			−1.04	0.13	−0.32	−1.30	-0.79	<0.001
Age			0.16	0.03	0.18	0.09	0.22	<0.001
Overcommitment			−0.37	0.13	−0.12	−0.62	-0.11	0.005
ER ratio			−1.87	0.83	−0.10	−3.50	-0.24	0.024

QOL: quality of life; R^2^: R squared; B: unstandardized coefficient; SE: standard error; β: standardized coefficient; CI: confidence interval.

As can be seen in Table 3, we used a forward stepwise regression analysis to determine variables independently associated with ER ratio, psychological morbidity and QOL. We found that female sex and being a nurse were positively associated with ER ratio, while age and working in reorganized hospital were inversely associated with ER ratio. For psychological morbidity, we found that life event, overcommitment and ER ratio were positively associated with GHQ (B = 0.84, 0.28, and 0.72; p = 0.007, < 0.001, and 0.005 respectively). Sex and history of hypnotic drug use were not significantly associated with GHQ (p = 0.087 and 0.07 respectively). For total QOL (additive from four QOL domains), total GHQ scores, age, overcommitment and ER ratio were identified as associated factors (B = −1.04, 0.16, -0.37, -1.87; p < 0.001, <0.001, 0.005 and < 0.001, respectively). With one-year increments of age, QOL increases by 0.16. With one-point increments in total GHQ score, QOL decreases by 1.04. Overcommitment and ER ratio were negatively associated with QOL.

## Discussion

In this study, we investigated the association between ERI, psychological morbidity and biological, psychological, social and environmental QOL in healthcare workers in military hospitals undergoing restructuring. We also analyzed the association between ER ratio, overcommitment and poor QOL. Women reported higher ERI scores, greater psychological morbidity and higher prevalences of poor physical and psychological QOL than men. We found that female sex, nurse occupation and the hospital undergoing accreditation ranking were positively associated with ER ratio. We also found that high ER ratio and overcommitment were associated with psychological morbidity and poor QOL in both sexes. Life events and ER ratio were positively associated with psychological morbidity. Age was positively associated with QOL; whereas, GHQ, overcommitment and ER ratio were negatively associated with QOL.

Reorganization was inversely associated with ER ratio, which may be explained by several reasons. First, the two merged hospitals had fewer patients, acute wards, and emergency cases, as well as lower surgery volumes. These factors will reflect a lower work load, which will undoubtedly lead to lower effort and lower ER ratios. Second, the larger hospital was undergoing accreditation ranking, which will increase both extrinsic and intrinsic effort among its employees. This would likely lead to a higher ER ratio. Third, the National Health Insurance in Taiwan, a health-access resource, and the Ministry of National Defense, which was under financial stress, implemented “the essence of the case” policy. Before and during the merge period, the preparation and reimbursement strategy announced by the Medical Affairs Bureau might decrease the hardship experienced by workers made redundant. Whatever the reason for high ER ratios among workers in the three hospitals, psychological morbidity and poor QOL were associated with job stress.

Although one study found no sex differences in reward [21], we found significant sex differences in reward in terms of social status control and total reward score, indicating that social gradient [7], employment grade levels, workplace culture and other factors should be considered. Social status control includes job insecurity and poor promotion prospects, as defined in the ERI model [11]. We used previously reported sex-specific aspects of work stress and health [2,24,25] to help us analyze sex differences in our data. In our study population, lower levels of education, alcohol use and smoking, and fewer leadership positions were noted among females. Younger ages, nurses, female sex and working in the accreditation hospital were positively associated with ER ratio and negatively associated with total QOL. A 1994 study of a Canadian national adult probability sample determined that high levels of job insecurity lowered self-rated health and increased distress and the use of medications, but had no impact on heavy drinking [26]. Differences between study findings may be because of different job categories in the study population and the social culture related to smoking and alcohol use in the military environment. Second, the accreditation taking place in Kaohsiung General Hospital lead to higher workloads, although the workers did not face job insecurity related to reorganization. Third, psychological morbidity might be a mediator between hypnotic drug use, job stress and total QOL. From our results, with are similar to those of Niedhammer *et al.*[2], we suggest that different guidance should be offered to help military healthcare workers balance ERI based on their job characteristics and hospital status (accreditation or reorganization).

This study found that overcommitment was associated with psychological morbidity and QOL in both sexes. This is in disagreement with the findings of other studies [8,27], where overcommitment was found to be more strongly associated with QOL in men than in women. Overcommitment at work has been associated with psychological morbidity in several studies [2,28,29]. We used ERI, a psychometrically robust measure of work-related stress and overcommitment that is grounded in sociological theory [22]. Overcommitment is seen as an independent concept because it stems from intrinsic effort/need for control and approval [30] and the inability to withdraw from work [31]. In our study, high overcommitment in males did not reveal a significant association with poor physical QOL, but did reveal a significant positive association with psychological morbidity. This may be because of the small sample size after stratification, or sex differences in coping levels. We hypothesize that intrinsic overcommitment, a personality trait possibly resulting in continued exaggerated efforts combined with disappointing rewards, might also increase the risk of poor health. In the future, we need a longitudinal study with a larger sample size to explore the role of overcommitment. Our multiple regression analysis also revealed that job category and reorganization status were associated with ERI, that ERI and overcommitment were associated with GHQ, and that ERI, overcommitment and GHQ were associated with QOL, suggesting the possibility that ERI and overcommitment may mediate and moderate the associations between psychosocial factors, GHQ and QOL. We found job insecurity and job category to have significant associations with health outcomes, as have other studies of ERI and health outcomes [2,19,32]. However, the relationships among these factors and mechanisms through which they exert their influence need further confirmation by other studies using path analysis.

To our knowledge, few studies have examined the relationship between job stress and the WHOQOL-BREF [1]. Unlike general health outcomes graded from one to eight by self-report, the WHOQOL-BREF is used to assess all domains of QOL [2]. It covers not only the physical and psychological domains of QOL, but also its social and environmental domains. Health-related outcome studies often use physical and psychological measurements while neglecting social and environmental outcome assessments [2,3,11,32,33]. Although there is a weak association between environmental QOL and job stress, the environmental domain remains an important issue at the workplace and in industrial health [1,34,35]. The social domain evaluates issues of personal relationships, sexual activity, practical social support and feelings of being respected and accepted. Adverse social relationships and job characteristics are associated with ill health [1,7,36]. Social support may act as a buffer and protect against the development of depression or anxiety in the face of poor working conditions [2,7,37]. However, this study did not find a significant association between the social QOL domain and ER ratio in either sex. This apparent lack of sensitivity may be because in the brief version, the social domain contains only four items, while in the original (WHOQOL-100), it contains twelve. One previous study suggested that sensitivity to change may be significantly reduced in the social domain of the WHOQOL-BREF, compared with that of the WHOQOL-100 [38]. Yao also suggested a need for further evaluation of the validity and reliability between the two instruments. When we analyzed the total scores of WHOQOL-BREF and GHQ to ER ratio and overcommitment by multiple regression analysis, mediating and moderating roles of ER ratio and overcommitment in the association between psychosocial factors, GHQ and QOL were noted [5]. Therefore, the WHOQOL-BREF is a useful alternative to the WHOQOL-100 when evaluating QOL in the physical, psychological and environmental domains. Researchers interested in measuring the social aspects of QOL are advised to use the lengthier WHOQOL-100.

The GHQ is used worldwide to measure mental health outcomes [1-3,39-41]. In many epidemiological studies, psychological morbidity was more prevalent in women than in men [1,2,6,40,42,43]. In this study, however, psychological morbidity was found to be more prevalent in both men and women who were overcommitted and reported higher ER ratios, especially in men. However, the regression analysis showed that female sex and nurse occupation were positively associated with ER ratio. In addition, female sex and ER ratio were positively associated with psychological morbidity. Despite the higher ER ratios in females than in males, the slightly higher prevalence of psychological morbidity in men may be due to information bias, as most of our male respondents were physicians (the group with the lowest response rate) and had higher educational levels, which have been associated with more willingness to express emotion [44,45]. Moreover, the physician job category often comes with more stress than other reported categories in non-healthcare related occupations [2,46]. Nevertheless, both male and female healthcare workers reporting overcommitment and high ER ratios had a higher prevalence of psychological morbidity.

This study has several limitations. First, the effective sample size was reduced after the data were divided into subgroups by sex, levels of ER ratio and overcommitment, and ERI groups. The statistical power may have been reduced for the small physician group. However, we used Sample size determination in health studies, version 2.0 (World Health Organization), selected the 95% confidence interval, anticipated a population prevalence of 0.025, population size of 50 000, and predicted the parameter of sample size to be 364. In this study, the population was 1269 and 791 workers returned the questionnaires, which was over the predicted sample size of 364. Our second limitation is that our study population were healthcare workers in hospitals, where many studies have found higher job stress and psychological morbidity [1,25,47-50], suggesting that the results cannot be generalized to the general population. This also means that the results may underestimate the relationship between job stress and health outcome, because a high turnover rate in healthcare workers has been noted in studies throughout the world. Those remaining at their jobs may be healthier than those who have left (the healthy worker effect). Third, the lower response rate from Kaohsiung General Hospital (especially from the other personnel who were older), which had a more balanced male:female ratio, higher work loading, and was undergoing accreditation ranking, compared with the higher response rates from the two merged hospitals, may have introduced selection bias. The accreditation process would increase the effort of healthcare workers in Kaohsiung General Hospital, while the merge would decrease the reward, and even lead to redundancies in the two merged hospitals. The ER ratio might be overestimated because of the low response rate from Kaohsiung General Hospital. High psychological morbidity and poor QOL might also be exaggerated. Fourth, because health outcomes and job stress were measured using self-report questionnaires, and higher response rates were found in merged hospitals, reporting bias could not be avoided. An overestimation of the association between job stress and QOL may be induced by information bias. However, the ER ratio involved intrinsic effort, which gave us some information about personality traits, suggesting that reporting bias did not change the association between job stress and health outcomes, as previous papers have also suggested [2,3]. Finally, the cross-sectional design of this study does not allow us to make conclusions about causal relationships between job stress and health outcomes. Psychological morbidity and poor QOL have been identified as potential outcomes of job stress in many follow-up studies [7,46,51].

## Conclusions

In conclusion, we found a clear association between ERI, psychological morbidity and poor physical and psychological QOL among the female healthcare workers in our study. In both sexes, we found that a high ER ratio and overcommitment were associated with poor QOL and psychological morbidity. However, no significant association was found between ER ratio and the social QOL domain in either sex, or the physical QOL domain in males. The impact of hospital reorganization and accreditation ranking on job characteristics and psychosocial factors, and the effects on healthcare workers should be considered more carefully by health policy makers when planning future policy.

## Competing interests

The authors declare none conflicts of interest.

## Authors’ contributions

DST contributed to the conception and design of the study, WCC contributed to the data collection process and assembling of data. CCL contributed to overlooking the data collection and data analysis. DST also contributed to the analysis of the data and further interpretation of the data. CYY was involved in the overall research process. All authors contributed to the drafting of the manuscript and approved of the final manuscript.

## Pre-publication history

The pre-publication history for this paper can be accessed here:

http://www.biomedcentral.com/1472-6963/12/309/prepub
